# Cardiac Magnetic Resonance Imaging to Determine Single Ventricle Function in a Pediatric Population is Feasible in a Large Trial Setting: Experience from the Single Ventricle Reconstruction Trial Longitudinal Follow up

**DOI:** 10.1007/s00246-023-03216-8

**Published:** 2023-07-05

**Authors:** Jon Detterich, Michael D. Taylor, Timothy C. Slesnick, Michael DiLorenzo, Anthony Hlavacek, Christopher Z. Lam, Shagun Sachdeva, Sean M. Lang, M. Jay Campbell, Jennifer Gerardin, Kevin K. Whitehead, Rahul H. Rathod, Mark Cartoski, Shaji Menon, Felicia Trachtenberg, Russell Gongwer, Jane Newburger, Caren Goldberg, Adam L. Dorfman, the Pediatric Heart Network Investigators

**Affiliations:** 1 Division of Cardiology, Children’s Hospital Los Angeles and the University of Southern California, 4650 Sunset Blvd MS34, Los Angeles, CA 90027 USA; 2 Department of Pediatrics, Heart Institute Cincinnati Children’s Hospital Medical Center, Cincinnati, OH USA; 3 Emory University School of Medicine, Atlanta, GA USA; 4 Children’s Healthcare of Atlanta, Sibley Heart Center Cardiology, Atlanta, GA USA; 5 Department of Pediatrics, Division of Pediatric Cardiology, Columbia University Irving Medical Center, New York, NY USA; 6 Division of Pediatric Cardiology, Department of Pediatrics, Medical University of South Carolina, Charleston, SC USA; 7 Department of Diagnostic Imaging, Hospital for Sick Children, Toronto, ON Canada; 8 Division of Pediatric Imaging, Department of Medical Imaging, University of Toronto, Toronto, ON Canada; 9 The Lillie Frank Abercrombie Section of Cardiology, Texas Children’s Hospital, Baylor College of Medicine, Houston, TX USA; 10 Duke University School of Medicine, Durham, NC USA; 11 Departments of Internal Medicine and Pediatrics, Children’s Hospital Wisconsin-Herma Heart Institute, Medical College of Wiscosin, Milwaukee, WI USA; 12 Division of Cardiology, Department of Pediatrics, Children’s Hospital of Philadelphia, Philadelphia, PA USA; 13 Department of Cardiology, Department of Pediatrics, Boston Children’s Hospital, Harvard Medical School, Boston, MA USA; 14 Division of Pediatric Cardiology, Nemours Cardiac Center, Nemours Children’s Hospital, Wilmington, DE, USA; 15 Division of Pediatric Cardiology, Primary Children’s Hospital, University of Utah, Salt Lake City, UT USA; 16 HealthCore Inc, Newton, MA USA; 17 Department of Pediatrics, University of Michigan Medical School, Ann Arbor, MI USA

**Keywords:** Cardiac Magenetic Resonance Imaging, Fontan Circulation, Pediatrics, Clinical Trial, Outcomes Research

## Abstract

The Single Ventricle Reconstruction (SVR) Trial was a randomized prospective trial designed to determine survival advantage of the modified Blalock-Taussig-Thomas shunt (BTTS) vs the right ventricle to pulmonary artery conduit (RVPAS) for patients with hypoplastic left heart syndrome. The primary aim of the long-term follow-up (SVRIII) was to determine the impact of shunt type on RV function. In this work, we describe the use of CMR in a large cohort follow up from the SVR Trial as a focused study of single ventricle function. The SVRIII protocol included short axis steady-state free precession imaging to assess single ventricle systolic function and flow quantification. There were 313 eligible SVRIII participants and 237 enrolled, ages ranging from 10 to 12.5 years. 177/237 (75%) participants underwent CMR. The most common reasons for not undergoing CMR exam were requirement for anesthesia (*n* = 14) or ICD/pacemaker (*n* = 11). A total of 168/177 (94%) CMR studies were diagnostic for RVEF. Median exam time was 54 [IQR 40–74] minutes, cine function exam time 20 [IQR 14–27] minutes, and flow quantification time 18 [IQR 12–25] minutes. There were 69/177 (39%) studies noted to have intra-thoracic artifacts, most common being susceptibility artifact from intra-thoracic metal. Not all artifacts resulted in non-diagnostic exams. These data describe the use and limitations of CMR for the assessment of cardiac function in a prospective trial setting in a grade-school-aged pediatric population with congenital heart disease. Many of the limitations are expected to decrease with the continued advancement of CMR technology.

## Introduction

The Single Ventricle Reconstruction (SVR) Trial was the first of its kind—a prospective randomized trial in congenital heart surgery designed to evaluate the effect of systemic to pulmonary shunt type on short-term survival [1]. The original aim of the SVR Trial was to compare transplantation-free survival in patients with single right ventricle (RV) anatomy, randomizing the patients into two groups, those who received a modified Blalock Taussig Thomas shunt (BTTS) and those who received a right ventricle to pulmonary artery conduit (RVPAS). In the original SVR Trial, early survival benefit was identified for the RVPAS group, but by one year of age, there was no difference in survival between the two shunt types [1]. The SVR cohort has now been studied in follow up work including SVR II [2], aimed at evaluating transplant-free survival following the Glenn and Fontan, and SVR III, the current long-term follow up evaluation of transplant-free survival and single ventricle function into adolescence. The current trial includes the opportunity to evaluate cardiac function in the single right ventricle.

Cardiac magnetic resonance imaging (CMR) is the gold standard for the assessment of right and left ventricular function and myocardial viability in congenital heart disease due to its superior blood-tissue contrast, 3-dimensional imaging, and quantification of blood flow, valve regurgitation, and chamber volumes [3, 4]. Despite the integration of structural and functional imaging into a comprehensive cardiac examination, CMR is viewed as an adjunctive imaging tool and its use in the pediatric population for prospective research has not been well described. A historic limitation to use of CMR prospectively for clinical trials has been the need for sedation or anesthesia in younger children. Clinical guidelines for CMR imaging are published, and many centers have performed CMR without sedation successfully in an increasingly younger population. Newer imaging techniques tailored to decrease scan times and reduce motion artifact, in combination with new child centered imaging environments that include CMR compatible video goggles and child life specialists, improve success rates for non-sedated pediatric CMR studies [5, 6]. The ability to perform CMR without sedation is an important consideration when planning a prospective study and aiming to minimize potential risk.

Given the inherent advantages of CMR for assessment of RV function, the SVR III trial was designed with right ventricular ejection fraction (RVEF) by CMR as the primary outcome measure. Taking into account the improvements over time in the use of CMR in younger children, this specific protocol was designed to overcome concerns regarding the feasibility of using CMR data as the prospective outcome of a clinical trial in 10–12-year-olds with hypoplastic left heart syndrome who have undergone Fontan operations. The objective of this report is to provide a detailed, modern assessment of the use and limitations of CMR in this prospective research setting.

## Materials and Methods

This study is a descriptive design to evaluate the use of CMR in the SVR long-term follow-up cohort using standard CMR methods [7]. The design and results of the original SVR study, supported by the Pediatric Heart Network (PHN), and the first follow up study, SVR II, have been previously published [1, 2]. There were originally 555 participants enrolled, with 275 in the BTTS group, 274 in the RVPAS group, and 6 exclusions. Transplant free survivors of the original SVR trial were eligible for enrollment in SVR III. The primary outcome measure was RVEF as measured by CMR. The Institutional Review Board of each participating center approved this study. The parents and participants provided informed consent and assent prior to participation.

As a part of the SVR III cohort study, participants were asked to undergo a non-contrast, non-sedated CMR exam to assess single ventricle function, blood flow in the major venous and arterial vessels including the superior vena cava (SVC), inferior vena cava (IVC)/Fontan conduit, right pulmonary artery (RPA), left pulmonary artery (LPA), neo-ascending aorta, descending aorta, pulmonary veins and tricuspid inflow. Exclusion criteria from CMR exam were the presence of an implantable device, including pacemakers and implantable defibrillators, or the need for anesthesia or sedation. Patients would still be enrolled in the SVR III study regardless of CMR study participation. If the participants underwent a clinically indicated CMR exam requiring contrast or sedation for clinical diagnostic purposes, and it included SVR III protocol components, it was used for analysis. The trial design, methodology, and complete SVR III protocol was published elsewhere. [8]

MRI protocol: The CMR protocol was designed by a small committee of representatives from participating PHN sites. All studies were performed on 1.5 T scanners (Phillips, General Electric, or Siemens). Participants were studied while freely breathing except as otherwise indicated. Scans were acquired with retrospective ECG gating, maximum parallel imaging acceleration factor of 2, and 3 signal averages. The use of a spoiled gradient recalled echo was encouraged in the setting of prominent susceptibility artifact. The CMR protocol was performed in a stepwise fashion, prioritizing SSFP cine data for measurement of RVEF, and included:

1. ECG gated 3 plane localizers
2. ECG gated balanced steady-state free precession (SSFP) cines: RV 2-chamber (vertical long axis), RV “4-chamber” (horizontal long axis), short axis stack through entire RV, and 3-chamber (outflow tract). Slice thickness 5–7 mm, pixel dimensions < 1.5 × 1.5 mm^2, TR < 4 ms, and TE ~ 2 ms.
3. Non-contrast MRA: 3D respiratory navigator and ECG gated SSFP acquisition from the mid-liver to top of the aortic arch with acquired resolution 1.5 mm^3. The trigger delay was optimized for the heart rate and the shot duration was maintained < 130 ms.
4. Phase contrast velocity acquisitions: SVC, IVC/Fontan, RPA, LPA, Neo ascending aorta, pulmonary veins, and tricuspid valve inflow

If CMR were performed as a clinically indicated exam and intravenous gadolinium contrast agent were used, additional data could include:

1. Contrast enhanced magnetic resonance angiography (MRA) from the liver to top of the aortic arch during an inspiratory breath hold.
2. Gradient echo inversion recovery late gadolinium enhancement (LGE) in the short axis stack, 2-chamber (VLA), 4-chamber (HLA), and 3-chamber (outflow tract) planes. LGE images were acquired with an expiratory breath hold if possible.
3. Flow analysis in other vessels not included in the standard research protocol above could also be included.

The anonymized CMR studies were transferred to the core laboratory via a commercial medical image cloud service (Ambra Health, New York). Before quantitative analysis study image quality was graded by the core laboratory on a 0–4 scale.

- 0-uninterpretable
- 1-poor: major artifacts compromising all data
- 2-fair: major artifacts compromising some data
- 3-good: minor artifacts without data compromise
- 4-excellent: no or minimal artifacts.

Study artifacts included missing slices, incomplete anatomical coverage, velocity aliasing, susceptibility effects due to intravascular devices and coils, patient motion, and ineffective cardiac gating. Images were determined to be adequate for analysis by two reviewers, and images had to be of a quality that allowed for endocardial and epicardial border identification over the entire ventricular mass and volume. Images were analyzed with CVI42 (Circle Cardiovascular Imaging, Calgary, Alberta). Image analysis included standard planimetry for ventricular volumes and ejection fraction and phase contrast velocity measurement. RV ejection fraction was the study primary outcome variable. If available, vessel diameter measurements were made on the non-contrast 3D SSFP MRA. DICOM header information was used to calculate the total scan, short axis function, and phase contrast velocity imaging times. Image time could not be calculated for some datasets due to the DICOM header information being removed during anonymization.

## Results

**Fig. 1:**
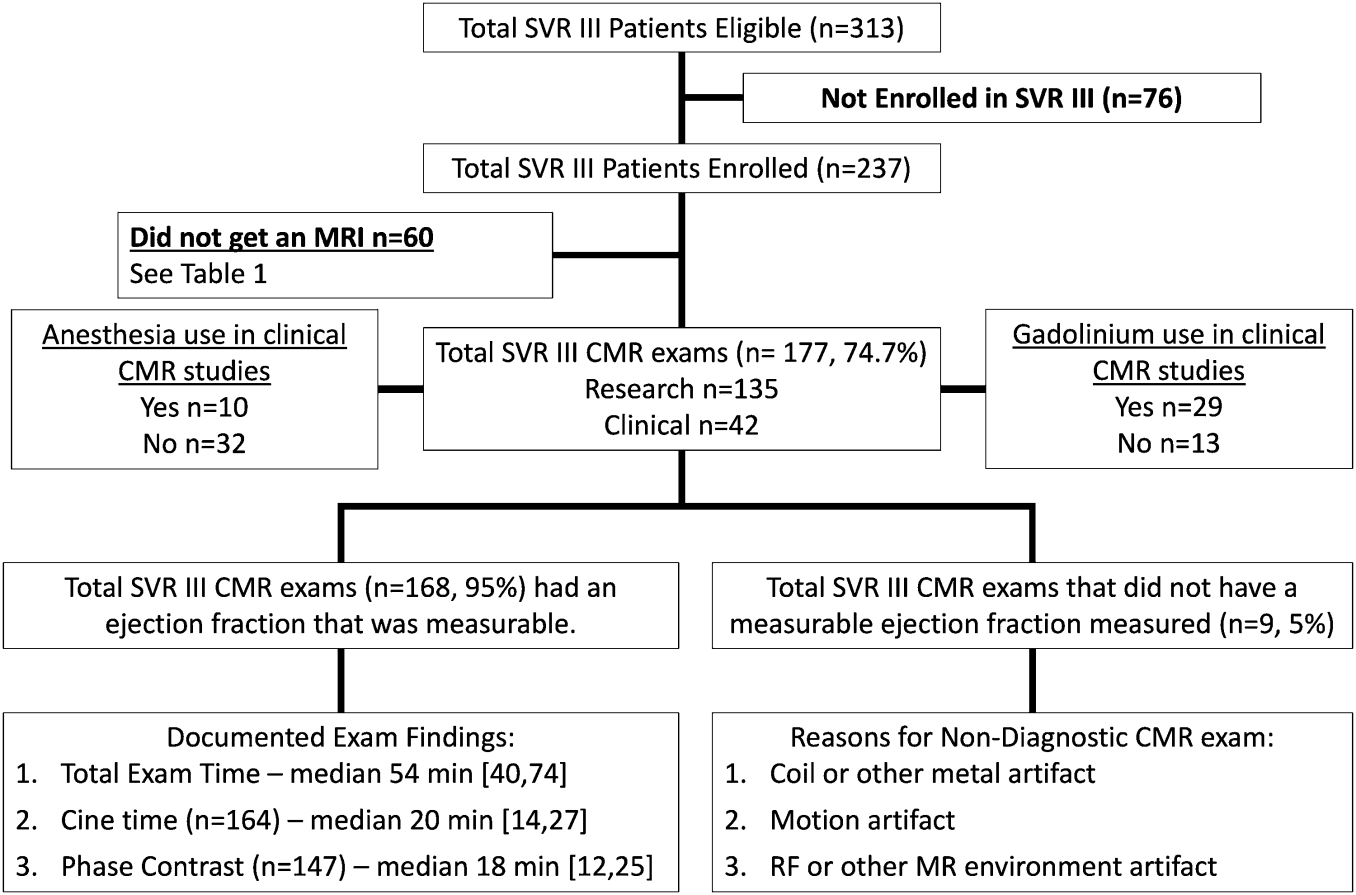
This is a flow chart demonstrating the breakdown of eligible patients enrolled in SVR III and the total number of CMR exams with adequate imaging for measurement of right ventricular ejection fraction

At the start of SVRIII, 313 patients were eligible for enrollment, and 237 patients enrolled with a median age of 10.76 years [IQR 10.28, 11.35] at the time of consent, Fig. 1. A total of 177/237 (74.7%) CMR studies were performed in participants with a median age of 10.90 [IQR 10.38, 11.45] at the time of CMR exam. Sixty patients did not have a CMR study, and the most common reason for not obtaining a CMR was the presence of a medical device that was not MRI safe (*n* = 19, 10 pacemaker/ICD). Other common reasons for not having CMR performed were the need for anesthesia (*n* = 14), refusal to undergo CMR, loss to follow up, claustrophobia, not able to complete prior to close of study, COVID-19 restrictions, and heart transplantation prior to obtaining CMR, Table 1.

**Table 1:** provides reasons enrolled participants did not undergo a CMR exam

Reason for not obtaining an MRI, *N* = 60	Frequenc*y*	%
Anesthesia Required	14	23.3
ICD/PPM/Device in place	11	16.7
Other medical device(s) or metallic fragments present	8	15
Not able to complete prior to close of enrollment	7	11.7
Refused	5	10
Claustrophobia/Anxiety	5	8.3
COVID 19 restrictions	5	8.3
Lost to follow-up	3	6.7
Heart Transplant prior to performing MRI	2	3.3

**Fig. 2:**
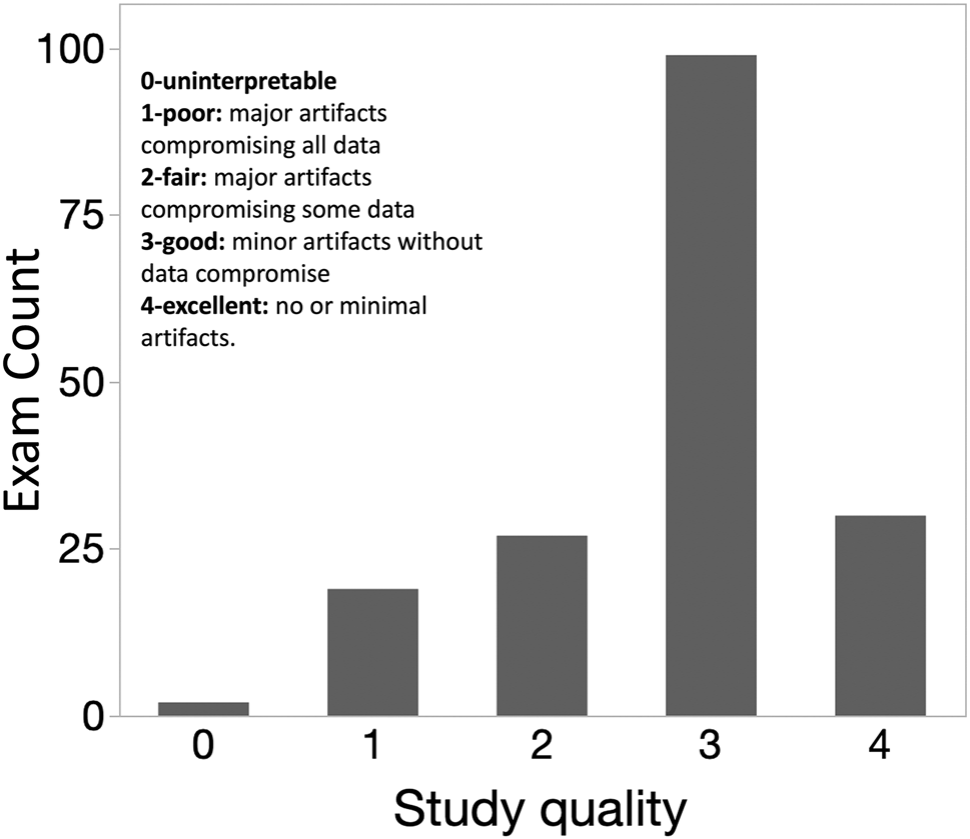
This figure demonstrates the distribution of study quality for all CMR exams performed in the SVR III study

The 177 CMR studies were performed at sites with scanners from three vendors: GE (18 exams), Philips (87 exams), and Siemens (72 exams). 135/177 (76%) studies were performed for the purpose of research only, without sedation or contrast. There were 42/177 (24%) studies performed for a clinical indication. There were 29/177 (16%) total studies performed using a gadolinium contrast agent. There were 10/177 (6%) total studies performed under generalized endotracheal tube anesthesia (*n* = 8) or iv sedation without intubation (*n* = 2). Therefore, 24 patients (10% of total enrollment, *n* = 237) either had a CMR under sedation/anesthesia or did not undergo CMR for research purposes due to the need for sedation/anesthesia. Most studies (98) were graded as “good” image quality and only 2 were graded as “uninterpretable” with another 7 that were graded as poor and could not measure the primary outcome, RVEF. The median image quality score was 3 [IQR 2, 3], Fig. 2. There were 168 studies (95% of those performed) of adequate quality to measure the RV ejection fraction. Table 2 lists the type of artifacts present.

**Table 2:** demonstrates the study quality grade and the type of artifacts noted in studies with that grade. The number in parentheses indicates the number of studies noted to have that artifact

Description	Grade	Type of Artifact
Uninterpretable	0 (*n* = 2)	Obliteration (2)
Poor	1 (*n* = 19)	coils, motion (2), bloom (5), incomplete imaging (4), error in images (2), Other Artifact (3), Obliteration
Fair	2 (*n* = 27)	Motion (4), PA Stent (2), bloom(6), phase contrast alias artifact, No Labels (3), limited PC images, inherent imaging problems, other (3)
Good	3 (*n* = 99)	Motion (4), PA Stent (5), Aortic Stent (2), Occluder (2), PC Aliasing, coil/bloom, no heart rate, PC artifact (3), No Labels, incomplete imaging, device artifact
Excellent	4 (*n* = 30)	Occluder, no PC images

The median total exam time was 54 min [IQR 40, 74]. There was high variability in the total exam time—minimum 8 min and maximum 154 min. The variability was secondary to sites adding LGE imaging or other pulse sequences to the base required exam, which is expected with the addition of imaging and contrast injection. Short exam times were from aborted studies or limited studies due to artifact. The median time to acquire adequate SSFP cine images for the primary outcome measure was 20 min [IQR 14, 27]. Similarly, the time for phase contrast image acquisition was 18 min [IQR 12, 25].

## Discussion

In the long-term follow-up of the Single Ventricle Reconstruction Trial [1] cohort, the primary outcome measure was RVEF as measured by CMR. The data presented currently assess the feasibility of obtaining this measure in the follow-up SVR population of 10 to 12.5-year-old patients with hypoplastic left heart syndrome status post Fontan surgery. CMR use as a research tool has been well described. However, few studies have provided a thorough review of the success and failure of obtaining CMR in a pediatric cohort, which is important for planning and powering prospective research. Furthermore, in a large cross-sectional study examining Fontan patients between 6 and 18 years of age with pre-determined exclusion criteria, only 159 patients (29%) underwent cardiac MRI out of 546 patients enrolled [3]. These data show CMR was successfully obtained in 75% of enrolled patients and single RV function was successfully obtained in 95% of those who underwent CMR. Exam times were short, and as a proportion of total enrollment, the need for anesthesia was infrequent. This study establishes baseline metrics for CMR as a safe and feasible functional outcome measure in pediatric clinical trials involving complex congenital heart disease.

Earlier data from the SVR Trial suggested that RV function as measured by echocardiography was impaired prior to Fontan surgery in patients who were palliated with a RVPAS versus those who received a mBTTS [9]. This raised the question of whether this surgical strategy, which includes right ventriculotomy in the neonatal period, could lead to long-term RV dysfunction and potentially worse Fontan outcomes. However, ventricular function in complex congenital heart disease, including the single RV, is best assessed by CMR due to its geometric independence for assessment of volumes and ejection fraction [3]. There is a paucity of published data utilizing CMR in a trial setting for measurement of ventricular function in elementary school-aged children with congenital heart disease, and it has been understood that school-aged children, particularly those with developmental impairments which are common for children with congenital heart disease, may struggle to cooperate with CMR examination due to the length of the study and the MRI environment [10–13]. Neurocognitive deficits are common in patients with single ventricle congenital heart disease with up to 30 to 40% of patients diagnosed with learning disabilities or having an individual educational plan [14, 15]. This is compared to only 5.9% of the enrolled participants who required anesthesia, suggesting that the high incidence of neurocognitive delays should not preclude the use of CMR in pediatric single ventricle research.

While 75% of the patients in this study were able to undergo CMR and nearly all of those had interpretable RV function data, 25% of the patients were unable to undergo CMR. The two most common reasons were MRI-unsafe implanted devices and need for sedation or anesthesia. Over time, the number of patients excluded from CMR studies for both of these reasons can be decreased with advances in device technology, CMR technique and the MRI environment.

Implanted devices are relative contraindications to CMR due to safety concerns and imaging artifacts. The presence of a cardiac implantable electronic device (CIED) was previously considered an absolute contraindication to MRI, but that has changed more recently. A growing body of literature in adults has demonstrated safety in scanning patients with non-MRI compatible devices [16], and there are data showing safety in children with congenital heart disease [17]. Newer MRI-conditional devices are also in use. In patients with hypoplastic left heart syndrome there is a 13% incidence of pacemaker placement by 8.5 years post Fontan [18]. In our study sample, 4.2% of enrolled patients had a pacemaker or ICD, and 7.6% had any device or metal fragment that prevented CMR exam. This lower incidence of pacemaker placement compared to published data may reflect a higher proportion of patients with pacemakers who were eligible to participate in SVR III, but did not enroll. This may also reflect a higher incidence of atrioventricular block in other forms of single ventricle CHD. Imaging susceptibility artifact leading to non-interpretable data remains a potential problem with CIEDs.

A recent single center review documented 20% anesthesia use in patients undergoing CMR exam for any reason at age 10 years [6]. In the current study of children 10 to 12.5 years of age, CMR could not be performed due to the need for anesthesia or sedation in 5.9% of subjects, a lower percentage than indicated in the earlier report. A higher incidence of developmental delay and/or behavioral difficulties in this patient population is a risk factor for need of sedation/anesthesia. While earlier work in this cohort demonstrated an increased percentage of participants at 6 years with scores in the at-risk or impaired range on measures of adaptive skills than expected for the population norms, there was a relatively low rate of SVR III participants who were unable to participate in the CMR due to lack of sedation/anesthesia. The lower percentage of subjects requiring sedation in this study could reflect advances in behavioral or CMR scanning techniques for helping children cooperate with non-sedated MRI [5]. These techniques, such as use of child life, movies and other distractions are anecdotally leading to decreased use of sedation/anesthesia for children in MRI at many centers.

CMR in younger children without anesthesia may require free breathing techniques, as many children are unable to comply with breath holding through the entire scan. Furthermore, children undergoing CMR under general anesthesia often require similar free breathing techniques to minimize the use of paralysis and ventilator pauses during an exam. While CMR more frequently required a breath hold for adequate imaging in the past, there are free breathing techniques in standard clinical use including multiple signal averaging and accelerated acquisition using parallel imaging, as well as emerging techniques such as real time CMR [19, 20] and four-dimensional imaging with cardiac registration [21–23]. As described in the methods, our protocol included accelerated image acquisition using parallel imaging and multiple signal averages for free breathing sequences. A total exam time of less than one hour and anatomic/functional assessment in 20 min is easily achievable. The vision for the future of CMR comprises rapid four-dimensional acquisitions with motion compensation, including in awake children less than 10 years of age and infants [21, 24], which will continue to break down barriers to CMR use in congenital heart disease research.

Study quality was adequate for measuring RVEF as the primary outcome using a free breathing CMR exam technique. Common artifacts experienced during the study included metallic susceptibility artifact, motion artifact, artifact from the MR environment (radiofrequency artifact) and phase contrast velocity encoding that was too low for the velocity of blood flow (aliasing). Older stainless steel coils and other implanted devices cause large susceptibility or “blooming” artifacts that obliterate much of the chest anatomy and were the most common reason for the inability to measure ejection fraction [25, 26]. Newer platinum, nitinol and alloy coils or stents cause no more than a local artifact and do not typically prevent the performance of quantitative measurements [27–30].

Our study is limited by potential selection bias in our long-term follow up of this specific cohort. Patients who were eligible but not enrolled may have been sicker, had developmental delays, genetic syndromes or had known implantable devices that resulted in the patients and families declining participation. There is also a survival bias in our follow up cohort, and it is possible that the patients who were transplanted or died could have had more difficult, lengthy, or poor-quality studies. This research protocol was designed for efficient CMR exams and not for evaluating accuracy or reproducibility of the volumetric analysis; however, it is important to note the protocol follows current guidelines for CMR use in children and adults with congenital heart disease 7. A minority of these studies were done for clinical indications that included contrast (16% of total studies) or sedation (5.6% of total studies), which often leads to lengthier examinations. These current data do not assess the ability of this patient population to tolerate longer, clinically indicated CMR exams without sedation or anesthesia.

## Conclusion

In this prospective, longitudinal follow up study of the single ventricle reconstruction trial participants, use of CMR as a primary outcome measure was successful in 71% of participants. Cine imaging necessary for measuring ejection fraction could be obtained in ≤ 20 min. The most common reasons for inability to obtain this measure were related to susceptibility artifact from metallic implants and inability to tolerate the exam without sedation or anesthesia. These data are useful for feasibility planning of CMR in pediatric clinical trials, which will likely improve with the use of non-ferrous implantable devices, changes in use of CMR for patients with CIEDs, and continued evolution of CMR towards shorter, motion compensated examinations.
